# Novel PNPLA2 gene mutation in a child causing neutral lipid storage disease with myopathy

**DOI:** 10.1186/s12881-018-0683-9

**Published:** 2018-09-17

**Authors:** Shouyan Zheng, Wei Liao

**Affiliations:** Department of Pediatrics, First Affiliated Hospital to Army Medical University, No. 30, Gaotanyan Street, Chongqing, 400038 China

**Keywords:** PNPLA2, NLSD-M, Gene mutation, Children

## Abstract

**Background:**

PNPLA2 gene mutations cause neutral lipid storage disease with myopathy (NLSD-M) or cardiomyopathies. The clinical phenotype, blood test results, imaging examination and gene analysis can be used to improve the understanding of NLSD-M, reduce the misdiagnosis rate and prevent physical disability and even premature death.

**Case presentation:**

We report a Chinese child with NLSD-M presenting with marked asymmetric skeletal myopathy and hypertrophic cardiomyopathy. Blood biochemical tests revealed increased creatine kinase levels, and echocardiography revealed a diffuse and thick left ventricular wall. Gene analysis revealed a homozygous mutation c.155C > G (p.Thr52Arg) in PNPLA2.

**Conclusions:**

An understanding of the characteristic features is essential for the early diagnosis of NLSD-M. Our data expand the allelic spectrum of PNPLA2 mutations, providing further evidence for genetic and clinical NLSD-M heterogeneity in younger individuals.

## Background

Neutral lipid storage disease with myopathy (NLSD-M) is an ultra-rare mostly autosomal recessive disorder caused by mutations in the PNPLA2 gene, which encodes adipose triglyceride lipase (ATGL). The condition was first reported in 2007 [1, 2]. To the best of our knowledge, only approximately 47 NLSD-M patients with PNPLA2 mutations have been reported to date, and the patients are all adults [3–13]. ATGL catalyzes the breakdown of triglyceride (TG) to diacylglycerol. ATGL dysfunction leads to accumulation of neutral lipids in multiple organs and tissues, including muscle, heart, liver and peripheral blood. The clinical features of NLSD-M include progressive myopathy, cardiomyopathy, hepatomegaly, diabetes, chronic pancreatitis, short stature and increased serum creatine kinase (CK) levels [14]. NLSD-M presents with skeletal muscle myopathy and severe dilated cardiomyopathy in approximately 40% of cases. However, given that NLSD-M is a rare metabolic disease, the pathophysiology of the disease is largely unclear, and genotype-phenotype correlations remain incomplete.

We herein report a Chinese child with NLSD-M presenting with marked asymmetric skeletal myopathy and hypertrophic cardiomyopathy (HCM) with a unreported homozygous mutation in PNPLA2. We report this case to improve our understanding of NLSD-M, reduce the misdiagnosis rate and prevent physical disability and even premature death.

## Case presentation

A 6-year-old Han Chinese boy had a 5-year history of progressive muscle weakness. His family first noticed he experienced difficulty in walking at approximately 1 year of age. As time progressed, he began to experience difficulties when climbing stairs and lifting heavy objects. He exhibited shortness of breath after activity and relief after rest. However, fever, headache, dizziness, palpitate, cough, sputum and convulsions were not observed. His previous medical history was unremarkable, and there was no family history of neuromuscular disease. His father died of a “work-related injury” five years ago, and his mother is now 40 years old without a significant medical history.

At the age of 4 years, he experienced difficulty in elevating his arms and walking, especially climbing stairs. He was admitted to the hospital with a potential diagnosis of “progressive muscular dystrophy”. The genetic analysis did not detect the 17 common mutation sites of DMD/BMD genes in his samples. Blood biochemical tests revealed increased CK levels of 1000 IU/L (normal range 0–225 IU/L). The doctor recommended he undergo further examinations, but his mother did not consent.

The patient’s condition continued to worsen. At 5 years of age, he visited our department because he would occasionally fall while walking and experienced shortness of breath after activity. CK levels were increased to 1617.00 IU/L (normal range 0–225 IU/L). Shortly after, he received consultation again due to echocardiography and electrocardiogram (ECG) abnormalities in the medical checkup. Medical examinations revealed the following: 1. Hypertrophic cardiomyopathy, 2. A small left ventricular measurement, 3. Lower left ventricular compliance in echocardiography and left ventricular hypertrophy with T wave inversion in ECG. On admission, his constitution indicated short stature without skin disorders, including ichthyosis or skin rash. He did not exhibit any ophthalmopathy or otopathy. He did not exhibit scoliosis or hyperlordosis but had Gower’s sign. His blood pressure was 98/60 mmHg, and his heart rate was 88 beats per minute. Cardiac auscultation revealed a normal rhythm.

Blood biochemical tests revealed increased CK levels of 1832 IU/L (normal range 0–225 IU/L). Routine blood tests revealed increased alanine transaminase (ALT) levels at 144 IU/L (reference range 0–42 IU/L), increased aspartate transaminase (AST) levels at 103 IU/L (reference range 0–42 IU/L), increased lactate dehydrogenase (LDH) levels at 1034 IU/L (reference range 114–240 IU/L), increased creatine kinase isoenzyme levels at 148 IU/L (reference range 0–25 IU/L) and normal triglyceride levels at 0.77 mmol/L (reference range 0.4–1.73 mmol/L). No abnormalities were observed in other routine biochemical tests.

The echocardiography revealed a diffuse and thick left ventricular wall. The left ventricular outflow tract was increasing rapidly, and the ejection fraction was 68%. Chest radiography revealed increased double lung texture, and the heart shadow was normal. The X-ray of bone age revealed that the maturity of the left hand bone was approximately 487 scores, which is equivalent to the age of a 4.8-year-old boy. A 24-h Holter-monitor recorded a sinus rhythm and an altered ST-T. Electromyography and evoked potential examination report revealed the following: 1. Normal movement conduction velocity (MCV) of the left median nerve, left ulnar nerve, left total nerve, and left tibial nerve. 2. Normal sensory conduction velocity (SCV) of the left median nerve, left ulnar nerve, left sural nerve, and left sural nerve. 3. F wave of left median nerve was normal. 4. Regarding H reflex, a normal H wave conduction velocity of the left tibial nerve was observed. 5. Electromyography revealed that the left gastrocnemius muscle and the inner muscle of the right femoral muscles were electrostatic. The mean time limit of the motion unit potential and the wave amplitude were in a normal range, and the electromyography of the examined muscle was normal. Abdominal ultrasonography indicated that the liver, biliary, pancreas, spleen, and kidney were normal. To improve our understanding of NLSD-M, reduce the misdiagnosis rate and prevent physical disability and even premature death, we recruited the child into a study and investigated PNPLA2.

**Fig. 1:**
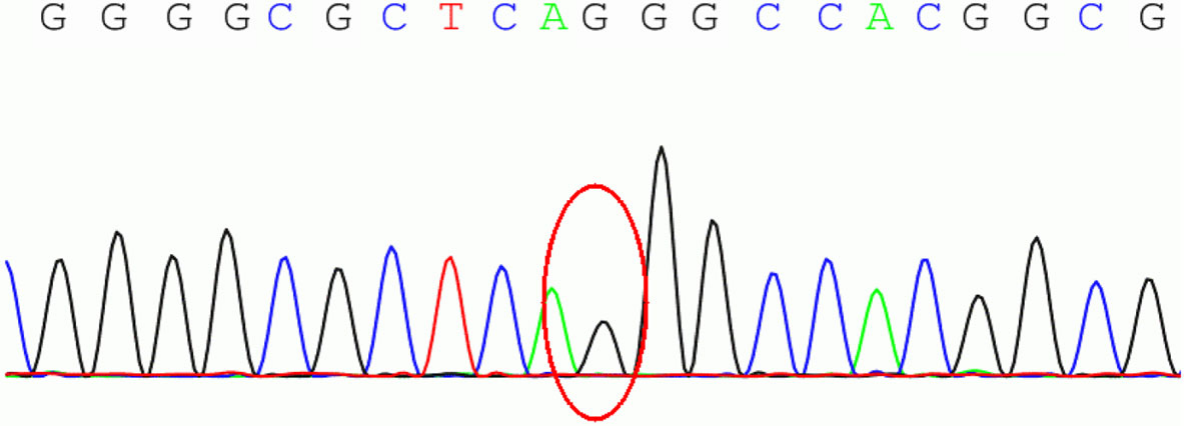
Genetic mutation in the PNPLA2 gene at chromosome 11:819873 of the patient

**Fig. 2:**
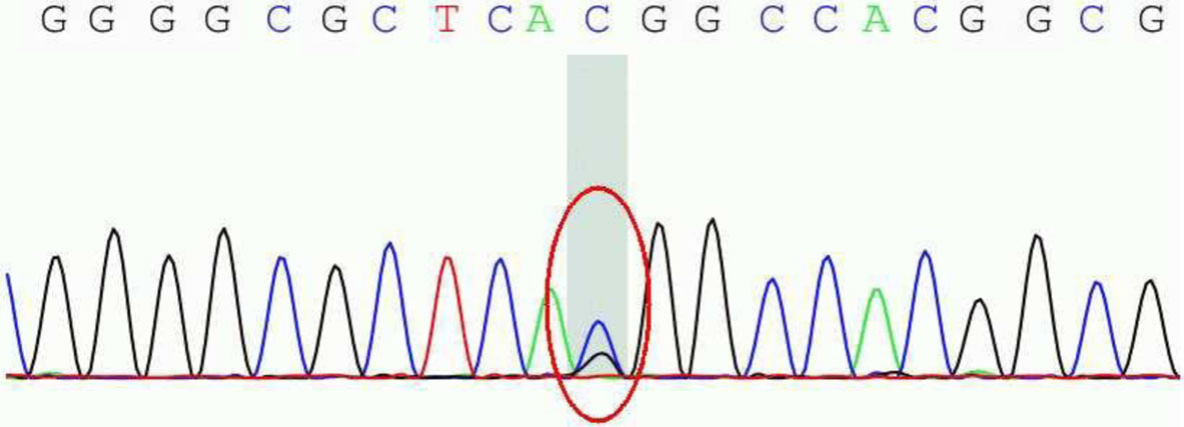
Genetic mutation in the PNPLA2 gene at chromosome 11:819873 of the patient’s mother

This study was approved by the Ethics Committee of First Affiliated Hospital to Army Medical University. Written informed consent for gene analysis was obtained from the legal guardian of the patient. We performed whole exome sequence capture on peripheral blood DNA samples from the patient. Sanger sequencing was used to validate the PNPLA2 (NM_020376: c.155C > G; p.Thr52Arg) homozygous variant identified by whole exome sequencing. Gene analysis revealed a novel homozygous mutation c.155C > G (p.Thr52Arg) in PNPLA2 (Fig. 1). Screening of the family confirmed that his mother was heterozygous for the PNPLA2 gene mutation (Fig. 2).

During hospitalization, his mother refused muscle biopsy for her child. This case was confirmed based on the clinical phenotype, blood test results, imaging examination and gene analysis. The patient was administered treatment with the phosphocreatine to protect the heart, and the symptoms were gradually relieved. He was subsequently discharged from the hospital. The follow-up revealed mild worsening of the condition and the development of muscle weakness of lower limbs. However, his heart function did not deteriorate. The patient remained fully ambulant during the follow-up period. He has exhibited no brain or peripheral nerve symptoms to date, and he has been able to continue attending school.

## Discussion and conclusions

In this report, we describe a 6-year-old patient with NLSD-M presenting with marked asymmetric skeletal myopathy and HCM secondary to a homozygous mutation c.155C > G (p.Thr52Arg) in PNPLA2. We also reviewed the clinical and genetic characteristics of NLSD-M cases in the literature. To our knowledge, NLSD-M onset is typically noted in adults. Reports about children with this disease are very rare. In this paper, the clinical data for a young patient with NLSD-M treated in our hospital were analyzed, and biochemical and genetic tests were performed to analyze the characteristics of NLSD-M. Lin et al. reported a missense mutation c.749A > C and a single-base deletion c.467del C in the PNPLA2 gene in three Chinese adults with NLSD-M [7].

NLSD-M is an ultra-rare disease. Its pathophysiology is largely unclear. The PNPLA2 gene encodes ATGL, a lipase that catalyzes the hydrolysis of triglycerides in mammalian adipose tissue. The results of biochemical studies revealed intracellular localization of ATGL combined with lipid droplets, but the catalytic activity was completely lost in the context of PNPLA2 mutations [15]. Some researchers have demonstrated that the cardiomyopathy phenotype is due to inability of the cardiac muscle to use free fatty acids, which are used in plasma rather than directly used for β-oxidization, and re-esterified triglycerides. In the presence of ATGL deficiency, re-esterified triglycerides accumulate in cardiomyocytes, causing hypertrophy and subsequent heart failure [16]. This notion may explain why mutations affecting the catalytic domain of the enzyme that lead to ATGL loss of function cause skeletal myopathy and cardiomyopathy. Here, we report a NLSD-M case with elevated CK levels detected during the course of the disease. Some reported cases were asymptomatic for myopathy presenting only elevated CK levels. Thus, more detailed studies are needed to elucidate the pathogenesis in the future.

The clinical progress of NLSD-M is slow and is primarily manifested as asymmetrical limb proximal or distal weakness, most of these have reported symptoms to be mild or indolent [17, 18]. The main clinical feature of NLSD-M is skeletal muscle myopathy, which is present in 100% of patients. Recent studies have demonstrated that gender may affect the clinical heart phenotype, even beyond the severity of PNPLA2 mutations. The presence severe PNPLA2 mutations is similar in men and women. Cardiac damage was reported in approximately 20% of NLSD-M female patients and 55% of male patients [12, 19]. According to the data, a gender difference in the phenotypic clinical expression in NLSD-M is noted. Males may die of heart failure before females despite presenting with HCM [19]. Thus, this patient should undergo long-term follow-up of heart function. In addition, the development of diabetes and pancreatitis should also be monitored in long-term follow-up.

An effective treatment or cure is not available at present. Enzyme replacement therapy may therefore become a viable treatment as noted in glycogen storage disease 2 [8]. In a human study, bezafibrate (a PPAR-α agonist) treatment in an individual with NLSD-M resulted in a marked reduction in cardiac and muscle fat content [20]. Some results showed that treatment with a β-adrenergic agent activates alternative triglyceride metabolism pathways in patients’ cells [6]. In this regard, additional larger clinical studies are needed to develop common therapeutic protocols.

In conclusion, we present a 6-year-old NLSD-M male patient with a PNPLA2 gene homozygous mutation. The patient exhibited myopathy and HCM. Our data expand the allelic spectrum of PNPLA2 mutations, providing further evidence for genetic and clinical NLSD-M heterogeneity in younger individuals. In addition, pediatricians should pay more attention to lipid myopathies, muscular dystrophies and myotonic dystrophies as a differential diagnosis for skeletal muscle weakness in the presence of increased CK levels and cardiomyopathies. The identification and study of PNPLA2 mutation is particularly important to help understand the disease mechanisms and develop more efficient and targeted therapies.

## Abbreviations

ALT: Alanine transaminase
AST: Aspartate transaminase
ATGL: Adipose triglyceride lipase
CK: Creatine kinase
ECG: Electrocardiogram
HCM: Hypertrophic cardiomyopathy
LDH: Lactate dehydrogenase
NLSD-M: Neutral lipid storage disease with myopathy
TG: Triglyceride
